# Prediction of brain metastasis progression after stereotactic radiosurgery: sensitivity to changing the definition of progression

**DOI:** 10.1117/1.JMI.12.2.024504

**Published:** 2025-04-08

**Authors:** Robert Policelli, David DeVries, Joanna Laba, Andrew Leung, Terence Tang, Ali Albweady, Ghada Alqaidy, Aaron D. Ward

**Affiliations:** a Western University, Department of Medical Biophysics, London, Ontario, Canada; b Verspeeten Family Cancer Centre, London Health Sciences Centre, Department of Radiation Oncology, London, Ontario, Canada; c Western University, Department of Oncology, London, Ontario, Canada; d Western University, Department of Medical Imaging, London, Ontario, Canada; e Qassim University, College of Medicine, Department of Radiology, Buraydah, Saudi Arabia; f King Fahad Armed Forces Hospital, Radiodiagnostic and Medical Imaging Department, Jeddah, Saudi Arabia

**Keywords:** brain metastases, machine learning, stereotactic radiosurgery, radiomics, magnetic resonance imaging

## Abstract

**Purpose:**

Machine learning (ML) has been used to predict tumor progression post-stereotactic radiosurgery (SRS) based on pre-treatment MRI for brain metastasis (BM) patients, but there is variability in the definition of what constitutes progression. We aim to measure the magnitude of the change of performance of an ML model predicting post-SRS progression when various definitions of progression were used.

**Approach:**

We collected pre- and post-SRS contrast-enhanced T1-weighted MRI scans from 62 BM patients (n=115 BMs). We trained a random decision forest model using radiomic features extracted from pre-SRS scans to predict progression versus non-progression for each BM. We varied the definition of progression by changing (1) the follow-up period (<9, <12, <15, <18, or <24 months); (2) the size change metric denoting progression (≥10%, ≥15%, ≥20%, or ≥25% in volume) or response assessment in neuro-oncology BM diameter (≥20%); and (3) whether BMs with treatment-related size changes (TRSCs) (pseudo-progression and/or radiation-necrosis) were labeled as progression. We measured performance using the area under the receiver operating characteristic curve (AUC).

**Results:**

When we varied the follow-up period, size change metric, and TRSC labeling, the AUCs had ranges of 0.06 (0.69 to 0.75), 0.06 (0.69 to 0.75), and 0.08 (0.69 to 0.77), respectively. Radiomic feature importance remained similar.

**Conclusions:**

Variability in the definition of BM progression has a measurable impact on the performance of an MRI radiomic-based ML model predicting post-SRS progression. A consistent, clinically relevant definition of post-SRS progression across studies would enable robust comparison of proposed ML systems, thereby accelerating progress in this field.

## 1 Introduction

Approximately 20% to 30% of all cancer patients will develop brain metastases (BMs) in their lifetimes^1^^,^^2^ and incidence is steadily rising. This rise could be attributed to higher imaging resolution and improved treatment modalities for primary cancers, resulting in longer patient survival times, and therefore, more time for primary tumors to metastasize to the brain.^3^ Although BMs are not usually the primary cause of death for cancer patients, they do have a substantial impact on the quality of life through chronic symptoms such as memory loss and loss of motor functions, and acute symptoms such as headaches and nausea.^4^ Patients therefore benefit from the early diagnosis and effective treatment of BMs.

One treatment option for BMs is stereotactic radiosurgery (SRS), which involves the precise delivery of high doses of radiation to BMs, typically in one or three fractions. SRS has become the standard of care for the treatment of a limited number of BMs given its excellent local control rates and minimal toxicity.^5^^,^^6^ Compared with more traditional whole-brain radiotherapy (WBRT), the major advantage of SRS is the improved sparing of normal brain tissue from radiation, resulting in fewer side effects including long-term neurocognitive changes.^5^^–^^7^

To measure the success of SRS, physicians monitor the treated BMs to determine if they continue to grow (progress) after the treatment.^8^ If a tumor does progress, the treatment is deemed a failure, whereas if the tumor decreases in size (regresses) or stops growing after treatment (remains stable), the treatment is deemed a success.^8^ To measure the growth or shrinkage of BMs post-SRS, physicians monitor the tumors using serial magnetic resonance imaging (MRI), though failure may take months to manifest.^9^ This long timeframe could lead to delays in future treatment options for these patients if there is treatment failure, resulting in the patient suffering from the side effects of both the BM and SRS. The accurate prediction of treatment failure prior to treatment could ensure these patients are spared from the potential side effects of SRS from the beginning, and instead allow them to explore other treatment options that could be more beneficial to them, such as systemic therapy, surgery, or WBRT.^7^ On the contrary, accurately predicting treatment success for SRS patients pre-treatment could ensure all patients who could benefit from this treatment receive it.

Machine learning (ML) models have been investigated in retrospective studies to attempt to predict the progression of BMs after SRS using pre-treatment imaging, with the goal to eventually eliminate the time required to evaluate post-treatment progression using conventional size change measurements.^10^ Many of these models have used radiomics, which aims to compute visual features within regions of interest (ROIs) in medical images, to differentiate between two or more outcomes.^10^ In the case of BMs, the ROI is the individual BM and the relevant outcome is BM progression or non-progression.^10^ Radiomic features can include descriptions of the shape and size of the BMs, voxel intensities, and patterns in the voxel intensities within the BMs known as texture features.^10^

Previous reports on the performance of ML models for the prediction of BM progression have substantial variability in the definition of progression.^11^^–^^22^ These variable definitions result in BMs being labeled as progression or non-progression in different ways. This complicates the comparison of studies and, potentially, the assessment of the relative performance of the studies’ proposed ML models. First, the definition of progression has varied in terms of how long after SRS each BM was monitored with imaging. This follow-up period ranged from as short as 3 months post-SRS to having no time limit.^11^^,^^12^ Other studies have varied the minimum required size change that denoted the progression of a BM. These studies either required a volume or diameter increase to indicate the progression of a BM. The thresholds of the minimum volume increase have ranged from 10% to 25% to denote progression.^10^^,^^13^ Diameter increases were usually denoted by a change in Response Assessment in Neuro-Oncology Brain Metastases (RANO-BM) diameter, which is a minimum of a 20% increase in the longest 2D nodal diameter of the BM to denote progression.^14^^–^^16^

One other way these studies varied the definition of progression was based on whether they accounted for BMs with treatment-related size changes (TRSCs). TRSCs are denoted by a perceived increase in tumor size that is not due to cancerous cell replication but is instead due to the side effects of radiation treatment.^17^ There are two subcategories of TRSCs. The first is known as pseudo-progression.^17^^,^^18^ This is denoted by a perceived increase in tumor size due to swelling around the tumor, which improves without further treatment, usually within 3–6 months of the initial treatment.^17^^,^^18^ The second category is known as radiation necrosis. This is also denoted on imaging as a perceived increase in tumor size but usually not presenting until after a time period of 6 or more months.^17^^,^^18^ Radiation necrosis is due to the death of cells and chronic inflammation that remain around the tumor after treatment and is less likely to resolve on its own.^17^^,^^18^ It can take multiple MR images over time for experts to distinguish between TRSCs and true progression, which is time-consuming.^19^ This makes it impractical in many contexts to make this distinction for every BM, even though the distinction could lead to different treatment routes for each patient. Various studies have either accounted for only radiation necrosis, only pseudoprogression, all TRSCs, or no TRSCs when predicting post-SRS progression, again making it difficult to determine the optimal ML model with this variation in the definition of treatment success.^12^^,^^20^^–^^22^

Ultimately, the successful clinical translation of an ML model to predict BM progression post-SRS will depend in large part on the demonstration of sufficient predictive performance on large, multi-center data sets, retrospectively and then prospectively. As the field moves toward this goal, it is important to be able to compare the relative performances of different reported ML models. This is already complicated by the fact that reported models are tested on different data sets. A further complication is the variability in the definition of the predicted progression endpoint. Because different definitions of “progression” affect the ground truth labels in the data set, it is expected that this would have some impact on the performance of a classifier. However, the magnitude of this impact is not known and needs to be known to determine the importance of standardizing a definition of progression for the purposes of predictive modeling. By understanding the effect of different definitions of progression on model performance, we can understand how models may vary when being tested with different ground truth labels from different centers. In this study, we sought to measure the magnitude of the impact on classifier performance, on the use of the different definitions of progression that have been reported in the literature. We aimed to do this by varying this definition of progression along different dimensions and measuring the difference in the reported performance of a state-of-the-art ML model for BM progression prediction.

We specifically aimed to examine:

a. How the performance of an MRI radiomic ML model to predict BM progression after SRS is affected by varying elements of the definition of progression, such as
  i. The follow-up period,
  ii. The size change metric,
  iii. The inclusion of TRSCs.
b. How the feature importance ranking by the same ML model is affected when predicting BM progression with varying definitions of progression.

## 2 Materials and Methods

### 2.1 Materials

Pre- and post-treatment T1-weighted contrast-enhanced (T1w-CE) MRIs were collected for 62 patients (n=117 BMs) from the Verspeeten Family Cancer Center (VFCC), London Health Sciences Centre in London, Ontario, Canada. These scans were collected for up to 2 years after treatment or until the date of death of the patient. This dataset includes all patients who were treated for brain metastases with SRS at VFCC between 2016 and 2022 with follow-up scans. The Western University Research Ethics Board approved this data collection. We excluded two patients with one BM each due to the BMs not having a measurable RANO-BM diameter on their pre-treatment MRI. This was due to the BMs being ring-enhancing cystic lesions with no visible nodule, making their diameter not measurable as per the RANO-BM guidelines.^15^ This exclusion was made to ensure all experiments that were executed would have the same dataset, ensuring the dataset was a constant variable throughout the study (n=115 BMs).

Treatment for these patients was conducted between 2016 and 2020 using a linear accelerator (linac)-based SRS at the VFCC in 1 to 3 fractions. The total planned dose ranged from 18 to 27 Gray (Gy) per BM. Treatment prescriptions and other patient demographics can be seen in Table 1.

**Table 1:** Clinical Information about the data set used in this study (n=115 BMs). BMs = brain metastases. This table was adapted from DeVries et al.^21^

Clinical information	Number of BMs
Sex of patient	
Female	70
Male	45
Primary cancer site	
Lung	88
Breast	10
Skin	8
Other	9
Prescription	
18 Gy in one fraction	7
20 Gy in one fraction	49
21 Gy in one fraction	2
24 Gy in three fractions	1
27 Gy in three fractions	56
Continuous clinical information	Median (range)
Age (years)	66.9 (43.6 to 82.2)
Initial BM volume (${\mathrm{mm}}^{3}$)	241 (13 to 14905)
Prescription dose (Gy)	21 (18 to 27)

The gross tumor volume (GTV) of each BM used to target the SRS was determined on the pre-treatment MRI by the treating physician manually based on the enhanced region on the pre-treatment MRI, including cystic regions if they were within the enhanced area. Radiologists also collected tumor measurements on all collected post-treatment scans. The measurements included the three orthogonal diameter measurements, collected by a neuroradiologist or neuroradiology fellow, of each BM (posterior-anterior, mediolateral, and superior-inferior).

They also included a measurement that adhered to the RANO-BM criteria, which measures the largest nodal diameter of each BM axially.^15^ TRSCs were assessed by one of four neuroradiologists, neuroradiology fellows, or radiation oncologists. They reviewed the tumors that had grown in diameter or volume at any point in the follow-up scans after SRS. They used all follow-up and pre-treatment MRI scans, available pathology reports, and records of salvage therapies to assess whether the tumor was truly progressing or appearing to grow due to pseudo-progression (short-term perceived increase in tumor size) or radiation necrosis (long-term, delayed perceived increase in tumor size).

### 2.2 Methods

For our experiments, the pre-treatment T1w-CE MRI was interpolated to an isotropic voxel size of $0.5\times 0.5\times 0.5\;{\mathrm{mm}}^{3}$ to ensure there was no variability in voxel size across patients’ MRI. We also applied Z-score intensity normalization to the MRI scans using the mean and standard deviation of the brain’s intensity values (not including the BMs) at three standard deviations to standardize intensity scaling across patients’ MRIs.

One hundred seven radiomic features were extracted from each BM’s pre-treatment T1w-CE MRI using PyRadiomics library v3.0.1 in Python v3.7.^23^ We extracted these features from each BM’s GTV as the defining ROI, including shape and size features, first-order texture features, and second-order texture features, for which we used 64 intensity value bins.

For all of our experiments, we trained a random decision forest ML model to distinguish between SRS success (stable or regressing BMs) and failure (progressing BMs) for each BM. Matlab 2019b v9.7.0.1471314 (The Mathworks Inc., Natick, United States) was used to run the experiments. Each experiment trained 250 random decision forest classifiers (250 iterations) with different training and testing splits of our dataset. Each training and testing split was determined by bootstrapping with resampling.^24^ The mean data splits for the experiments were 36.2% [95% confidence interval (CI): 35.5% to 36.9%] of data in the testing set (unseen by the training set), and 63.8% (CI:63.1% to 64.5%) of data in the training set. We used a Bayesian hyperparameter optimizer on our training dataset prior to training in each iteration to find the optimal hyperparameters. To ensure there were not more variables than data points in our model, the hyperparameter for tree depth was set to not be larger than the amount of data points in our training dataset. The mean tree depth and number of trees for each experiment can be seen in Figs. 6 and 7 of the Appendix. We used an inter-feature correlation filter prior to each iteration of model training to remove features that had a correlation coefficient >0.8 and would therefore be redundant. The random decision forest classifier used the selected features to classify between progressing and non-progressing BMs. A depiction of which features were used and not used in each experiment can be found in Fig. 8 of the Appendix. A flow diagram of our model can be found in Fig. 9 of the Appendix.

We measured the performance of our models by calculating the area under the receiver operating characteristic (AUC) curve on each iteration of testing. We then averaged the AUCs across all iterations to obtain an overall model performance and calculated the associated 95% confidence interval.

We also measured performance by calculating experiment sensitivity and specificity on each iteration of testing. To calculate these metrics, we found the optimal operating point for model performance (AUC) on each iteration’s training set. This was done to ensure were not overestimating our model performance by taking the maximum sensitivity and specificity of the test set. With this operating point from the training set, we then found the sensitivity and specificity of the testing data of each iteration of the experiment at this operating point. We found mean sensitivity and specificity with a 95% confidence interval for each experiment.

Finally, we measured model performance by calculating the area under the precision-recall curve (AU-PRC).^25^ Although most published studies attempting to predict BM progression do not use this metric, AU-PRC is commonly used to measure ML model performance when there is a class imbalance.^25^ We also calculated the baseline AU-PRC (number of progressing BMs divided by the total BMs in the dataset).^25^ An AU-PRC above the baseline indicates that the model is performing with an AU-PRC at a rate better than random chance.^25^ We calculated the AU-PRC of each iteration of our experiments and found the mean AU-PRC of each experiment.

### 2.3 Performance Analysis

**Fig. 1:**
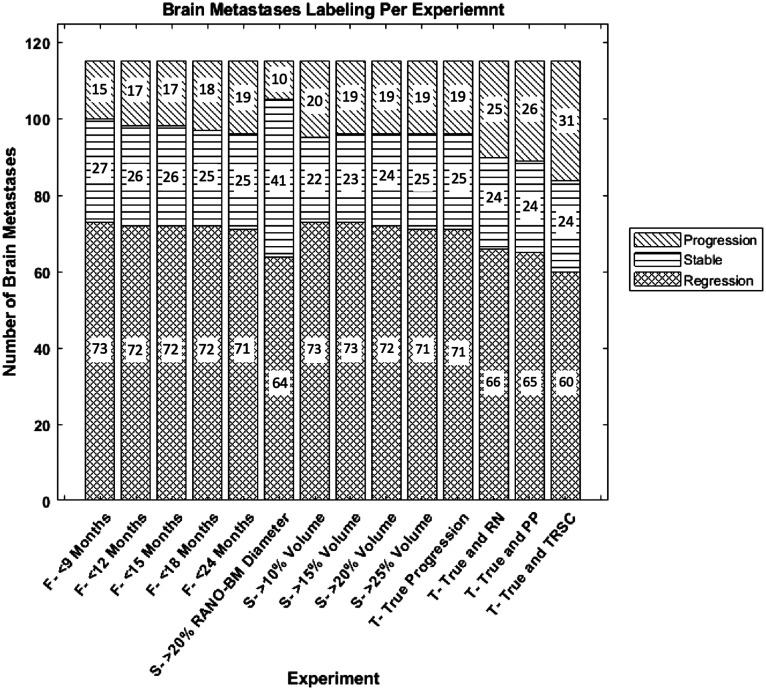
Labeling of brain metastases as progressing, stable, or regressing for each experiment. The x-axis label denotes the criteria for each brain metastasis to be called as progression. The numbers within the plot denote the number of BMs with each label per experiment. For our experiments, non-progression = stable + regression BMs. True = true progression, RN = radiation necrosis, PP = pseudo-progression, TRSC = treatment-related size changes. F = follow-up period category, S = size change metric category, and T = treatment-related size change category.

For all experiments, the criteria to label a BM as treatment failure (progression) is outlined in Table 2, and a more in-depth description of the criteria is provided in the following paragraphs. Our experimental design aimed to be reflective of what is currently done in the literature to measure the impact of the definition of progression and did not have the initial goal of producing the best model possible. The labeling breakdown of BMs in each experiment as progression or non-progression (stable and regressing BMs) can be seen in Fig. 1.

**Table 2:** Description of the definition of progression for each experiment. All BMs that meet the criteria of the follow-up period, size change metric, and treatment-related size changes (TRSC) are labeled as treatment failure (progression) for that experiment. RN = radiation necrosis and PP = pseudo-progression.

Experiment	Follow-up period	Size change metric	TRSC
Progression follow-up period			
<9 months	< 9 months	>25% volume	True progression
<12 months	<12 months	>25% volume	True progression
<15 months	<15 months	>25% volume	True progression
<18 months	<18 months	>25% volume	True progression
<24 months	<24 months	>25% volume	True progression
Size change metric			
>20% RANO-BM diameter	<24 months	>20% RANO-BM diameter	True progression
>10% volume	<24 months	>10% volume	True progression
>15% volume	<24 months	>15% volume	True progression
>20% volume	<24 months	>20% volume	True progression
>25% volume	<24 months	>25% volume	True progression
Treatment-related size changes			
Progression = true progression	<24 months	>25% volume	True progression
Progression = true progression + RN	<24 months	>25% volume	True progression + RN
Progression = true progression + PP	<24 months	>25% volume	True progression + PP
Progression = true progression + RN + PP	<24 months	>25% volume	True progression + RN + PP

The first variable in defining progression versus non-progression we changed between experiments was the length of the follow-up period after SRS during which the BMs were monitored. We looked at follow-up periods of 9, 12, 15, 18, and 24 months. Although four previous studies used follow-up periods that were less than 9 months,^11^^,^^14^^,^^16^^,^^26^ not all of our patients had follow-up completed prior to 9 months, so we chose 9 months as the shortest follow-up period for our study. Our study followed patients for 2 years post SRS, so we therefore set our longest follow-up period to 24 months.

The second variable we changed between experiments was the size change metric used to label each BM as progression or non-progression. The first type of size change metric used in the literature is measuring diameter increases in the BM to denote progression. The most common diameter measurement is known as RANO-BM, which is a minimum of a 20% increase in diameter.^15^^,^^27^ This is a measurement used on a per-patient basis, but it has been adapted to be used on a per-BM basis as has been done in studies by Gutsche et al. and Karami et al.^16^^,^^21^ The RANO-BM criteria also denote that any tumor less than 10 mm in size must have a minimal increase of 3 mm in RANO-BM diameter to denote progression due to uncertainty in small measurements on MRI, so we also abided by that in our labeling of each BM.^13^ Again, consistent with the RANO-BM criteria, any follow-up diameter measurements that could not made due to their cystic nature were also excluded.

The second type of size change metric used in the literature is a minimum volume increase of a BM to denote progression. We used minimum volume increases of 10%, 15%, 20%, and 25% to cover the full range of what has been used in the literature.^10^^,^^13^^,^^20^^,^^22^^,^^28^ Because the follow-up T1w-CE MRI scans we retrospectively collected did not have contoured BMs, we had to estimate the volume of each BM. To estimate the volume of each BM after SRS, we used the three orthogonal diameter measurements of each BM (posterior-anterior, mediolateral, and superior-inferior) and took the product of them. This was done in the same manner as in DeVries et al.^10^ and Kawahara et al.^28^ To again reduce the uncertainty of small tumor measurements on MRI, we similarly used the RANO-BM minimum diameter increase of 3 mm to adapt our volume measurements. We used this 3 mm value in 3 dimensions to get a minimum volume change of $27\;{\mathrm{mm}}^{3}$ compared with the pre-treatment volume estimates to denote the progression of a BM, which was also done in the study by DeVries et al.^10^

The third variable we changed between experiments was the inclusion of TRSCs when labeling progressing BMs. Again, the clinicians used the MRI scans, the pathology reports, and the records of salvage therapies to assess whether the tumor was truly progressing or appearing to grow due to pseudo-progression or radiation necrosis. These assessments were then used to run experiments that called only true progressing BMs as progression, true progressing and pseudo-progressing BMs as progression, true progressing and radiation necrosis BMs as progression, and all TRSCs (pseudo-progression and radiation necrosis) and true progression called as progression. These combinations have been used in previous ML studies.^12^^,^^20^^–^^22^

For these three experiment categories, we held the other two variables constant throughout all experiments. When varying the follow-up period, we chose a volume size change threshold of greater than 25% to denote BM progression for these experiments. This was selected arbitrarily based on a common size change metric in the literature.^10^^,^^29^ We also accounted for all TRSCs in this data set, to ensure that these BMs were not labeled as progression. This was selected because it only accounts for BMs that truly progressed. When varying the size change metric, we chose to have a maximal follow-up period of 24 months after SRS and similarly accounted for all TRSCs. This follow-up period was selected based on being the longest one we have. When varying the TRSC inclusion, we again chose to have a volume size change threshold of greater than 25% to denote BM progression for these experiments and a maximal follow-up period of 24 months after SRS.

One final type of progression classification we tested was attempting to predict treatment failure as progressing and stable BMs compared with treatment success as only BMs that regressed in size. All results and descriptions of these experiments can be seen in the Appendix.

### 2.4 Feature Analysis

In each ML experiment, we collected feature importance for the model to classify between progressing and non-progressing BMs. Random decision forest models supplied feature importance scores based on the extent to which a feature helps to distinguish between progression and non-progression. The feature importance scores were collected for each iteration of the experiment and then normalized between 0 and 1 after each iteration. All features removed by the correlation filter were given a feature importance score of zero. The feature importance scores were averaged across all experiment iterations and then renormalized between 0 and 1 again to obtain feature importance scores for each experiment.

**Fig. 2:**
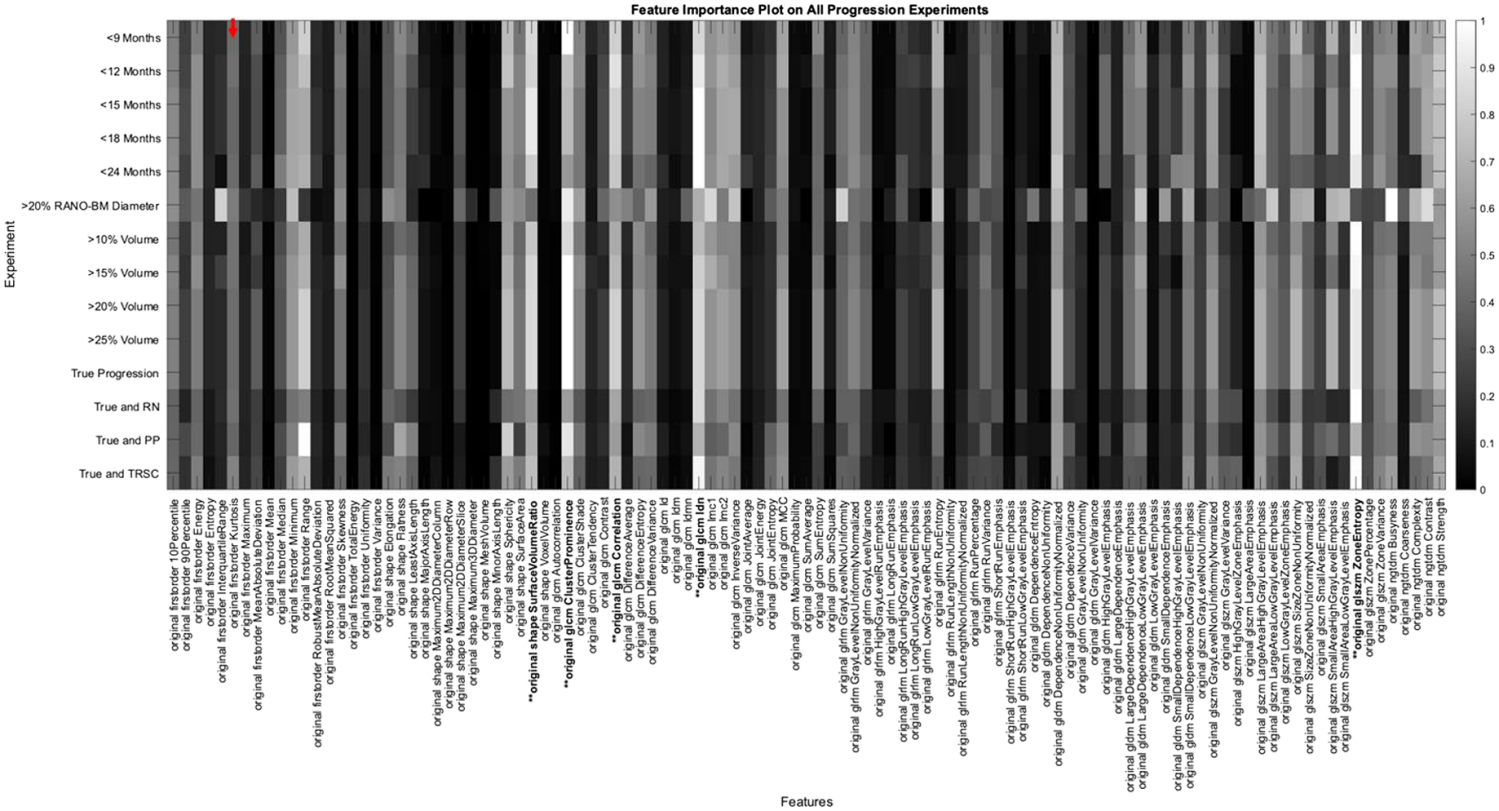
Depiction of the importance of each feature for each experiment. The row indicates that experiment is being referenced, and the column indicates what feature is being measured. Based on the color bar on the right of the image, the value of 1 (white color) indicates the feature was the most important for that experiment. The value of 0 (black color) indicates the feature was the least important for that experiment. For example, the red arrow near the top left of the figure points to the number of times the feature “original first order Kurtosis” was selected in the experiment that used a 9-month follow-up period. This point on the color bar indicated that the Kurtosis feature importance was 0.418, demonstrating this feature was more important than 41.8% of all the features for the classification of progression in the 9-month follow-up period experiment.

For this study, we wanted to compare how the feature importance scores varied between each individual experiment. To do this, we averaged the feature scores from each experiment category, which can be seen graphically in Fig. 2. We also wanted to measure how the feature importance scores varied for each of the three experiment categories that we conducted: follow-up period experiments, size change metric experiments, and TRSC experiments. We did this to attempt to determine if the change in model performance was due to the features being prioritized by the model, or if not, what features were important for a model to classify between BM progression and non-progression. The code used to generate the experiments can be freely accessed on GitHub.

## 3 Results

### 3.1 Performance Analysis

**Fig. 3:**
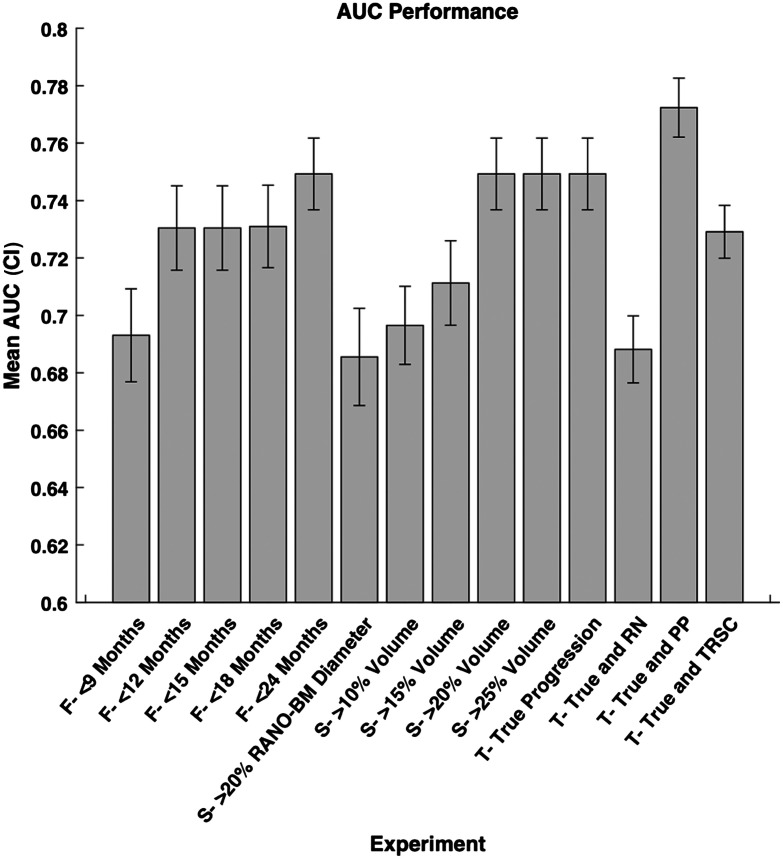
Mean model performance in terms of AUC for each experimental configuration we used. The 95% confidence interval of each experiment is shown by the lines on the graph. CI = confidence interval, AUC = area under the receiver operating characteristic curve, True = true progression, RN = radiation necrosis, PP = pseudo-progression, and TRSC = treatment-related size changes. F = follow-up period category, S = size change metric category, and T = treatment-related size change category.

**Fig. 4:**
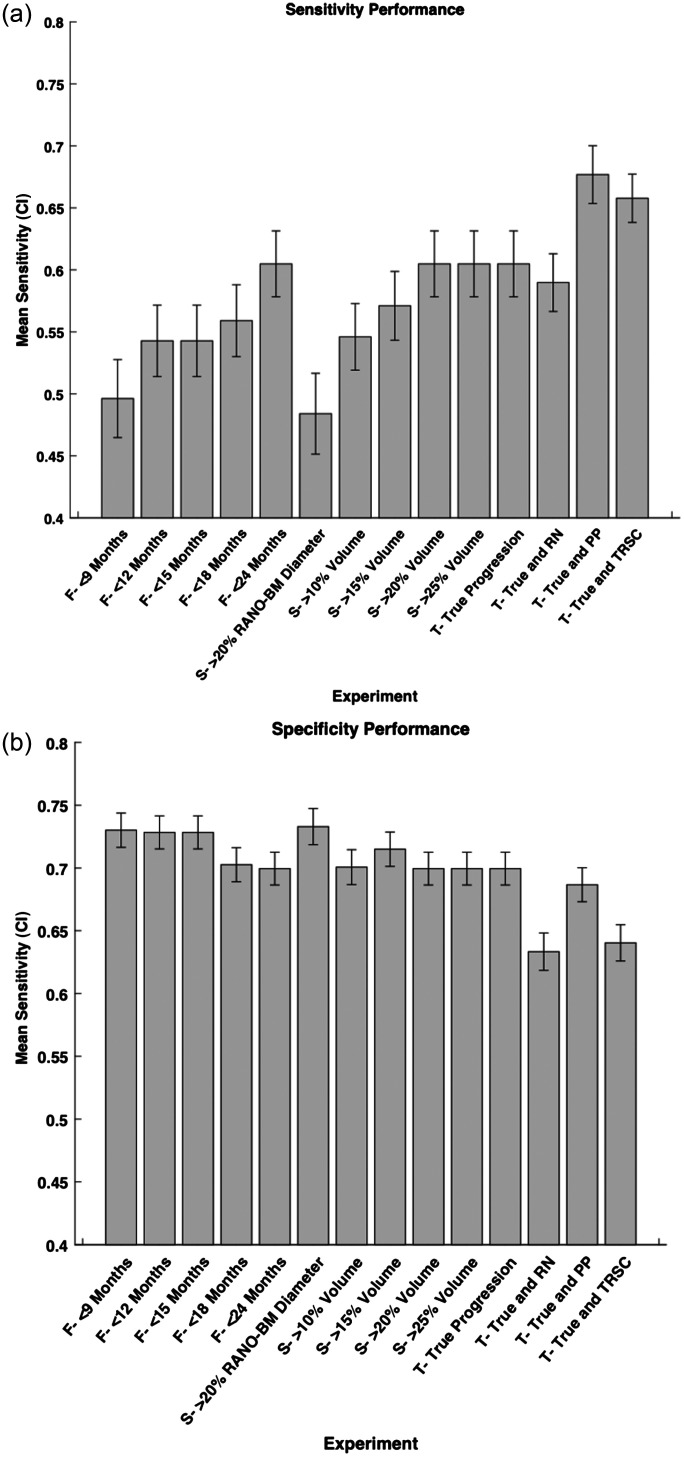
(a) Sensitivity and (b) specificity of each experimental configuration we used. The 95% confidence interval of each experiment is shown by the lines on the graph. CI = confidence interval, True = true progression, RN = radiation necrosis, PP = pseudo-progression, and TRSC = treatment-related size changes. F = follow-up period category, S = size change metric category, and T = treatment-related size change category.

We calculated the model performance for each definition of progression defined previously in the methods (Fig. 3). We found that the variation in follow-up time after SRS in predicting progression resulted in an AUC range of 0.06 (0.69 to 0.75). We also found the variation in the size change metric after SRS in predicting progression resulted in an AUC range of 0.06 (0.69 to 0.75). Finally, we found the variation in using TRSCs after SRS in predicting progression resulted in an AUC range of 0.08 (0.69 to 0.77). The AUC range over all experiments was also 0.08. We also found that the difference in sensitivity was 0.20, and the difference in specificity was 0.10 between the highest and lowest performing models of each metric, respectively (Fig. 4). Therefore, changing each variable in the definition of progression has a measurable effect on model performance when attempting to predict progression after SRS in terms of AUC, sensitivity, and specificity.

**Fig. 5:**
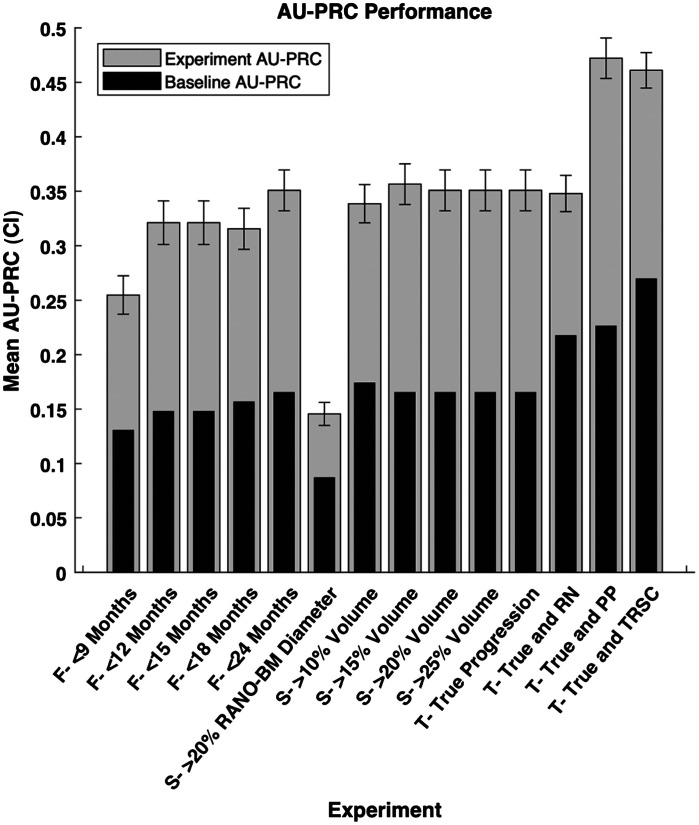
Model performance in terms of AU-PRC for each experimental configuration we used. The 95% confidence interval of each experiment is shown by the lines on the graph. CI = confidence interval, AU-PRC = area under the precision-recall curve, True = true progression, RN = radiation necrosis, PP = pseudo-progression, and TRSC = treatment-related size changes. F = follow-up period category, S = size change metric category, and T = treatment-related size change category.

To combat our class-imbalanced dataset, we also calculated AU-PRC for each experiment defined in the methods (Fig. 5). The range of AU-PRCs when changing the follow-up period, size change metric, and TRSC inclusion were 0.10 (0.25 to 0.35), 0.22 (0.14 to 0.36), and 0.12 (0.35 to 0.47). Therefore, changing the definition of progression also has an effect on the model AU-PRC.

Because most of our dataset contains brain metastases from patients with their primary tumor in the lung, we calculated AUC, AU-PRC, sensitivity, and specificity on BMs originating from the lungs compared with the other primary cancer sites. These data can be found in Table 4 in the Appendix and demonstrated that the lung data were not biasing model performance.

### 3.2 Feature Analysis

We also analyzed how the important radiomic features varied when changing each variable to predict progression. The most important features for each of the three experiment categories and overall experiments can be seen in Table 3. The five most important features were the same when altering each experiment category, though their order of importance changed. The important features also matched the five most important features over all experiments (Table 3). These five most important features included four higher-order texture features and one shape and size-based feature. A depiction of the most important features for each experiment predicting progression can be seen in Fig. 2. The five most important features in the experiment categories were found to be stable between experiments as well (indicated in boldface and preceded by ** in Fig. 2).

**Table 3:** Ranking of the most important features for each experiment category in the study. The score indicates the normalized importance of the feature for the set of experiments, with a score of 1 indicating the most important, and a score of 0 indicating the least important feature. GLSZM = gray level size zone matrix, GLCM = gray level co-occurrence matrix, Idn = inverse difference normalized.

Rank	Follow-up period		Size change metric		Treatment-related size changes		All	
	Feature	Average score	Feature	Average score	Feature	Average score	feature	Average Score
1	GLSZM zone entropy	0.99	GLCM cluster prominence	0.98	GLSZM zone entropy	0.94	GLSZM zone entropy	0.92
2	GLCM Idn	0.93	GLSZM zone entropy	0.85	GLCM cluster prominence	0.88	GLCM cluster prominence	0.88
3	Shape surface volume ratio	0.89	GLCM Idn	0.80	GLCM Idn	0.86	GLCM Idn	0.86
4	GLCM	0.86	Shape	0.80	Shape	0.81	Shape	0.84
	Correlation		Surface volume ratio		Surface volume ratio		Surface volume ratio	
5	GLCM cluster prominence	0.79	GLCM	0.75	GLCM	0.75	GLCM	0.79
			correlation		correlation			
							correlation	

## 4 Discussion

Our study is the first to analyze how varying the definition of progression impacts the magnitude of performance and feature selection of a model to predict BM progression post-SRS using MRI radiomics. Our findings indicate that varying the definition of progression has a material impact on the performance of the random decision forest MRI radiomic model predicting progression. This defined impact on model performance could be used to encourage collaboration between centers to use a standard definition of progression so that future models can potentially have less performance variability across centers. Moreover, our experiments reveal the consistent importance of certain radiomic features, in conjunction with the random decision forest model, in distinguishing between progressing and non-progressing BMs.

### 4.1 Performance Analysis

Our model performance (AUCs) had a range of 0.69 to 0.75 for follow-up periods between 9 and 24 months, which demonstrated that that follow-up period does have an impact on how an MRI radiomic model performs when predicting progression post-SRS. Other studies looking to classify post-SRS BM progression reported an AUC of 0.78^20^ using a 12-month follow-up period and AUCs between 0.72 and 0.95 for follow-up periods with no time constraint.^12^^,^^13^^,^^22^^,^^26^^,^^30^ Similar to our findings, the best model performance (AUC = 0.95) occurred when there was a longer follow-up period.^22^ That being said, without consistent definitions of the size change metrics and TRSCs in the studies mentioned above, the comparison of model performances based on follow-up period is difficult to make. Our experiments help overcome this issue by keeping all variables constant except for the follow-up period and demonstrate that a shorter follow-up period correlates with worse model performance.

The increase in model sensitivity from 0.48 to 0.60 as the follow-up period was lengthened also demonstrated that the follow-up period impacts the ability of an MRI radiomic model to detect progressing BMs. The specificity on the other hand decreased from 0.73 to 0.69 as follow-up time increased. This indicates that the model worsens its detection of non-progressing BMs as it improves its detection of progressing BMs with a longer follow-up period. The improvement of model AUC and sensitivity with a longer follow-up period could be due to the fact that having more time to monitor a patient may lead to more accurate progression diagnoses. In some cases, the BM does not start progressing until many months after SRS, so labeling a tumor as stable after a few months could be incorrect if the tumor progresses afterward. The ability to use more follow-up scans over time to create a more complete picture of BM growth produces more accurate predictions using our techniques. Therefore, using as many follow-up scans as possible or setting a minimal follow-up time could be a technique that is considered when attempting to arrive at a consensus on a definition of progression going forward.

The range of model performance (AUC) from 0.69 to 0.75 when varying the size change metric demonstrated that varying the size change metric also produced practically significant differences in our model’s performance. Previous reports of post-SRS progression model performance based on using RANO-BM measurements to define progression demonstrated AUCs between 0.74 and 0.93,^11^^,^^14^^,^^16^^,^^21^^,^^27^ and studies using other volume change metrics to define progression produced AUCs between 0.78 and 0.95.^10^^,^^13^^,^^20^^,^^22^^,^^26^^,^^29^ Although a study by Du et al. reported an AUC of 0.93,^7^ they used multiple MRI pulse sequences and ROIs from within and around the tumor, making their study challenging to compare with ours and many others which exclusively used T1w-CE MRI scans and ROIs within the tumor. Because our goal was to measure the impact of the definition of progression on model performance and not necessarily to produce the best-performing model, we designed our experiments to be consistent with most published studies in the literature.

We found that using a definition of progression based on volume changes resulted in enhanced model AUC and sensitivity, compared with the AUC and sensitivity achieved when using diameter changes to define progression. This trend was not seen for specificity. We speculate that this improvement in model AUC and sensitivity occurred because volume change is a more complete, three-dimensional representation of tumor size change, whereas the RANO-BM measurement only measures tumor size changes in two dimensions. The study by Ocaña-Tienda et al. demonstrates that volume is more effective at identifying BM progression compared with RANO-BM measurements, concordant with our findings.^31^ We also hypothesize that pre-treatment image phenotypes are more strongly correlated to large tumor volume changes because our best model performance occurred with a volume change of >25% and the worst model performance occurred with a volume change of >10%, but this needs to be tested on a larger data set going forward.

Finally, the range of AUCs between 0.69 and 0.77, sensitivity between 0.60 and 0.68, and specificity between 0.63 and 0.70 when varying how TRSCs were labeled in our data set also demonstrated how TRSC variation resulted in a measurable change in model performance. In the literature, AUCs ranged from 0.74 to 0.95^12^^,^^20^^–^^22^ when varying the definition of TRSCs included in their models, but again, comparisons are limited due to other confounding variables not being held constant between studies. The study by Liao et al. (AUC = 0.95) had a large testing set (n=93) for their model, but their model only used the pre-treatment MRI and the latest follow-up MRI to label each BM as progression or non-progression.^22^ This method of labeling each BM prevents the reader from seeing the trends in BM size change over time, not allowing the reader to call cases as potential TRSCs. This lack of TRSCs inclusion could have resulted in the model predicting some BMs with TRSCs instead of only progressing BMs, which have different retreatment options and make it difficult to compare this model with other models that accounted for TRSCs. Again, the differences in the definition of progression between models emphasize the need for a more consistent definition of progression going forward to eventually have these models implemented in the clinic.

When calculating AU-PRC for the same experiments, we found similar trends to the AUC values we calculated. When changing the follow-up period, we similarly noticed an increase in model AU-PRC as the follow-up period lengthened. When varying the size change metric, we saw the worst AU-PRC for the RANO-BM criteria, similar to our AUC results. Finally, when changing TRSCs, we similarly saw that this variable has a measurable effect on model performance. For all experiments, we demonstrated that the AU-PRC was greater than the baseline AUPRC, indicating that even with an imbalanced dataset, the model is still correctly detecting progression at a rate better than chance.

The findings in this study could potentially motivate the organization of a grand challenge, where many researchers test their model on the same dataset with the same definition of progression to compare model effectiveness accurately. Without a grand challenge, performance differences of around 0.08 could be potentially attributed to different definitions of progression between datasets, so this could help eliminate this potential source of variability when comparing the performance of different approaches.

### 4.2 Feature Analysis

The feature importance analysis demonstrated the stability of the most important features in our random decision forest models while the definition of progression was varied. The gray-level size-zone matrix (GLSZM) zone entropy and gray-level co-occurrence matrix (GLCM) inverse difference normalized (IDN) features are both measures of homogeneity of the GLSZM and GLCM matrices, emphasizing co-occurrence matrix homogeneity as an important feature to analyze going forward. GLCM cluster prominence is a measure of the skewness of voxel intensities in the GLCM matrix, indicating the importance of skewness for this classification problem. A high GLCM correlation, which we saw in many of the progressing BMs’ pre-treatment MRI, indicates there was a wide range of gray level values throughout the BM, but adjacent voxels had only small differences in gray levels, concordant with GLCMs having strong response along the diagonal. Finally, shape and size were also found to be important in discerning between progressing and non-progressing BMs through the surface area to volume ratio feature selected in our study. The consistency of the features being selected when altering the definition of progression also suggests the potential robustness of the random decision forest model for this problem, but this remains to be tested on unseen data in future work.

Some variations in feature importance can be seen in Fig. 2 for the RANO-BM size change metric experiment and some TRSC experiments. We hypothesize that the RANO-BM size change metric criteria caused the largest change in the labeling of the progression of those experiments and therefore changed, which features were deemed to be the most significant. The TRSC experiments had the same five most important features as the other two categories of experiments, indicating that the features that differed in importance between experiments were features that had less of an effect on model performance.

Other studies have also revealed the important features selected by their models attempting to predict SRS progression. Jalalafar et al. reported on a model that selected CE-T1w MRI GLCM IDN as an important feature.^30^ The study by Liao et al. also found that GLCM correlation was a predictor of SRS outcome in their model.^22^ Other studies also found GLCM correlation and GLCM IDN were important features in their model, but on FLAIR, T2w, or diffusion-weighted MRI instead of T1w MRI scans.^14^^,^^16^^,^^20^^,^^22^^,^^30^ All of these features are concordant with the features used by our model. The similarity of some features between models in the literature, even with the variability in experimental design, provides evidence for the importance of these radiomic features to distinguish between progressing and non-progressing BMs more generally.

### 4.3 Limitations

Our study has helped to highlight the importance of a standard definition of progression, but it is important to acknowledge some limitations. First, the study relies on imaging and SRS outcome data from a single center with a limited sample size, which could impact the generalizability of this study to other institutions. We also have a class imbalance in our dataset, which would impact the ability of AUC to be an effective performance metric. We combat this limitation by measuring the same effect on precision-recall curves.

We also realize that the majority (76.5%) of our BMs arose from patients with lung cancer. We tested this limitation by calculating performance metrics for only BMs from lung cancer patients and BMs from other primary cancer sites to demonstrate that the lung patients were not biasing model performance.

We also recognize that we have only examined the effect of the variability of the definition of progression on one type of model (random decision forest). Although the single model may also limit the generalizability of the study, holding the model constant was a crucial aspect of our experiments. The singular model type was used to ensure all other variables remained constant when changing the definition of progression to compare the variable changes effectively.

## 5 Conclusion

Variability in the definition of BM progression used in published studies has a measurable impact on the performance of a T1w-CE MRI radiomic-based ML model predicting post-SRS progression. Variability in the progression definition caused AUCs measuring the predictive accuracy of the models to vary by as much as 0.08. This variability in AUC occurred despite the most important radiomic features being consistent across the different experimental conditions. This study highlights the need for a consistent, clinically relevant definition of post-SRS progression across studies to enable robust comparison of proposed ML systems, potentially even through a grand challenge, thereby accelerating progress toward the development of a BM post-SRS progression predictive model that can be translated into clinical practice.

## 6 Appendix

**Fig. 6:**
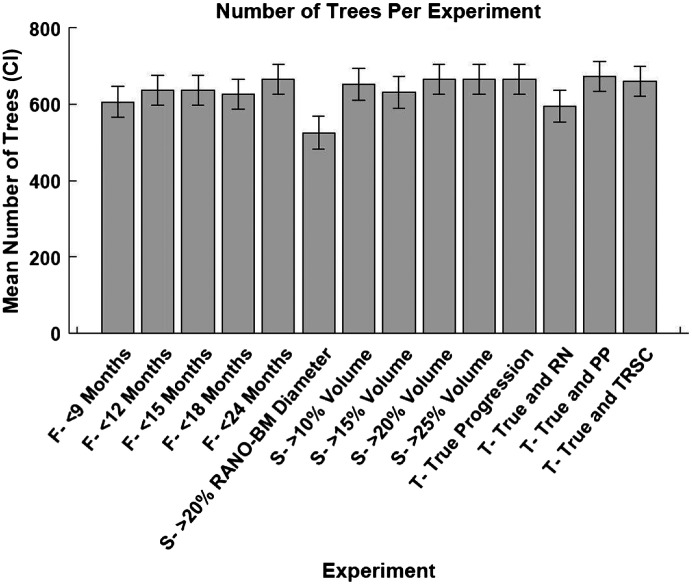
Mean number of trees optimized for each experiment in the study. The 95% confidence interval of each experiment is shown by the lines on the graph. CI = confidence interval, True = true progression, RN = radiation necrosis, PP = pseudo-progression, and TRSC = treatment-related size changes. F = follow-up period category, S = size change metric category, and T = treatment-related size change category.

**Fig. 7:**
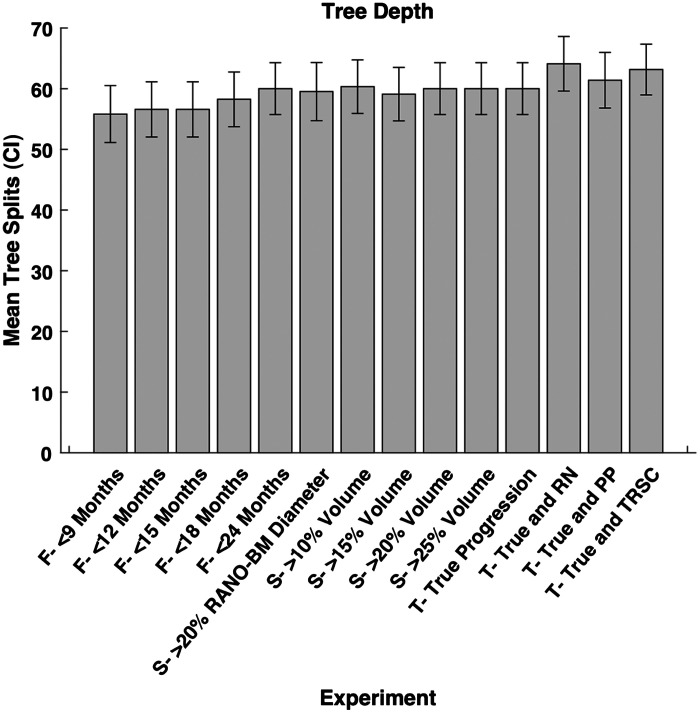
Mean depth of each tree optimized for each model in the experiments for this study. The 95% confidence interval of each experiment is shown by the lines on the graph. CI = confidence interval, True = true progression, RN = radiation necrosis, PP = pseudo-progression, and TRSC = treatment-related size changes. F = follow-up period category, S = size change metric category, and T = treatment-related size change category.

The average depth of the trees and the number of trees in each experiment performed can be seen in Figs. 6 and 7, respectively. The depth of each tree was set to be optimized between 1 and the number of data points in the training set. The number of trees was optimized to be between 10 and 1000 trees for each experiment.

**Fig. 8:**
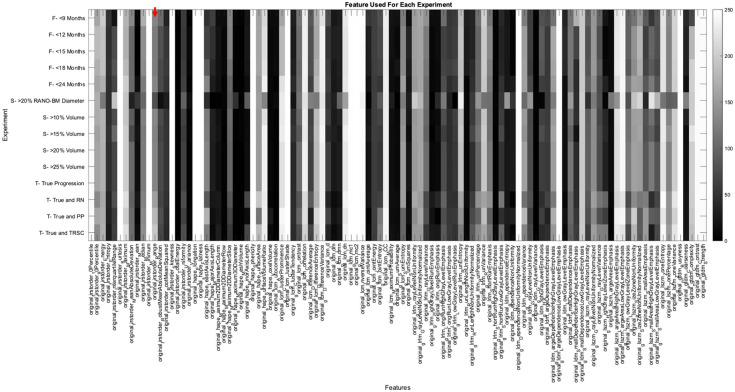
Depiction of the number of times each feature was selected for each experiment. The row indicates which experiment is being referenced, and the column indicates what feature is being measured. Based on the color bar on the right of the image, the value of 250 (white color) indicates the feature was selected by the correlation filter feature selector in every iteration of the experiment. The value of 0 (black color) indicates the feature was not selected at all for the respective experiment. For example, the red arrow points at the number of times the feature “original first-order range” was selected in the experiment that used a 9-month follow-up period. This point on the color bar indicated that this feature was selected and used in 130 iterations of the experiment.

**Fig. 9:**
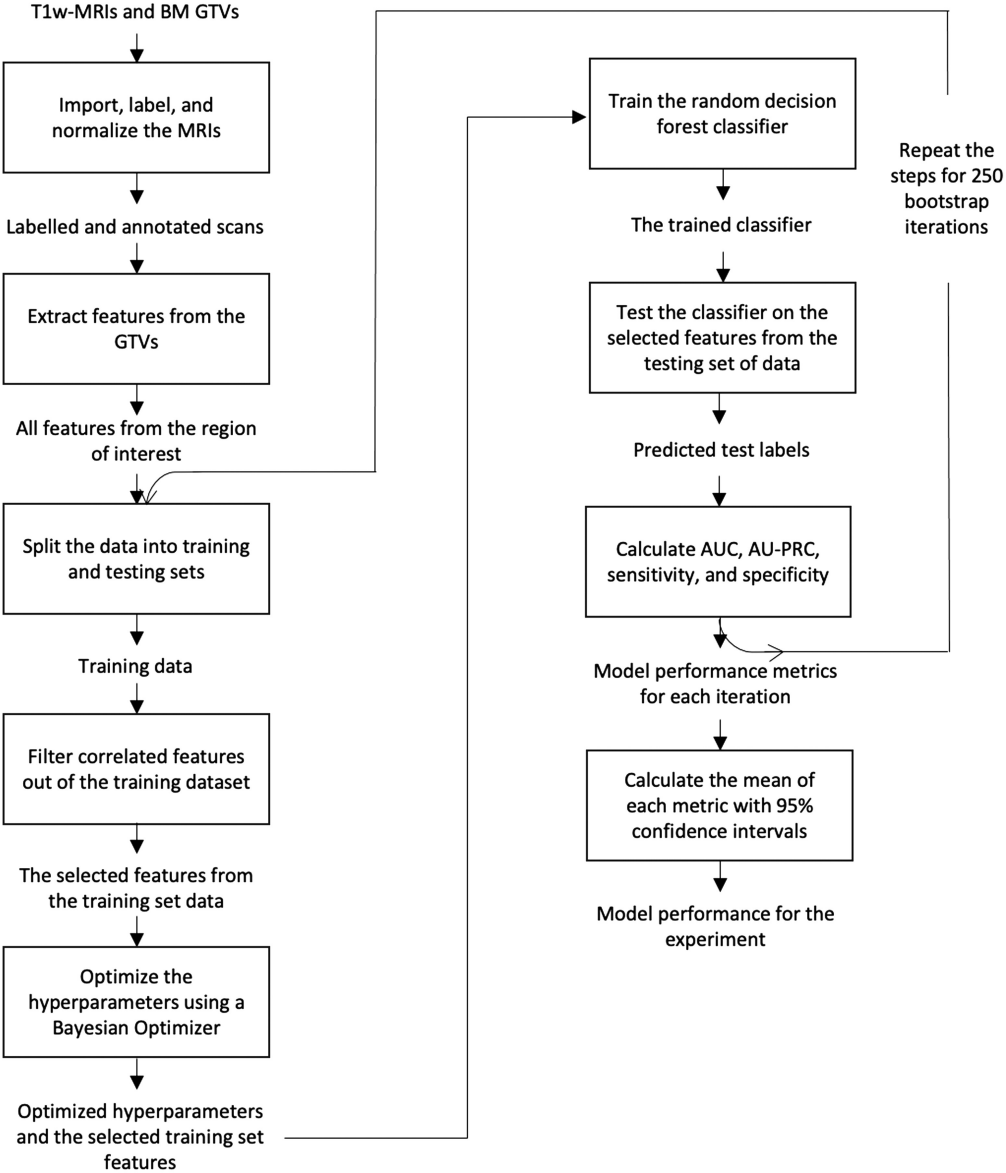
Flow chart depicting the machine learning-based radiomic model. MRI = magnetic resonance imaging, GTV = gross tumor volume, AUC = area under the receiver operating characteristic curve, and AU-PRC = area under the precision-recall curve.

The number of times a feature was selected to be used in each experiment can be seen in Fig. 8. The features were chosen prior to training in each bootstrap iteration using the training set data. The selected features were deemed to not be more the 80% correlated with other features in the training set. A depiction of our model in a flow diagram can be seen in Fig. 9.

We also tested the performance of our models on patients with primary cancer in their lungs versus other primary cancer sites. This was completed to test whether the primary cancer site affected the performance of our model because our dataset consisted of mainly patients with lung cancer as their primary cancer site. We calculated AUC, AU-PRC, sensitivity, and specificity, for both primary cancer site groups and displayed the data in Table 4. To calculate these metrics, we gathered all testing set progression probabilities over all bootstrap iterations and grouped them by lung or other primary cancer site. The aggregated probabilities for both cancer site groups were used to calculate the error metrics. This aggregated method was used because some of the “other” primary cancer site groups had a small number of BMs (n=27), so training models for this group were not feasible. This analysis demonstrated that the lung data was not biasing model performance. For the size change metric experiments, the AUC range for the patients with primary lung cancer was between 0.60 and 0.74, while for patients with other primary cancer sites, it ranged from 0.57 to 0.70, demonstrating a similar range of AUC performance as a function of changing this metric.

One last type of progression classification that we tested was attempting to predict treatment failure as progressing and stable BMs (whereas in the previous experiments, only progressing BMs were recorded as treatment failures) compared with treatment success as only BMs that regressed in size (whereas in the previous experiments, regressing and stable BMs were recorded as treatment successes). For purposes of comparison with other reported studies, we defined treatment failure/success in this manner,^32^ but this classification has less clinical relevance due to the fact that progressing BMs usually result in a reassessed treatment plan, whereas stable BMs are often treated similarly to regressing BMs with continuing the current treatment or monitoring of the disease.^33^ We therefore included this investigation in the Appendix.

**Table 4:** Mean AUC, AU-PRC, sensitivity, and specificity for each experiment comparing primary cancer sites of the lungs and other sites. The 95% confidence interval of each experiment is shown in square brackets. CI = 95% confidence interval, True = true progression, RN = radiation necrosis, PP = pseudo-progression, and TRSC = treatment-related size changes. F = follow-up period category, S = size change metric category, and T = treatment-related size change category.

Experiment	Lung (n=88 BMs)				Other sites (n=27 BMs)			
	AUC [CI]	AU-PRC [CI]	Sensitivity [CI]	Specificity [CI]	AUC [CI]	AU-PRC [CI]	Sensitivity [CI]	Specificity [CI]
F- <9 months	0.67 [0.65 to 0.68]	0.23 [0.23 to 0.24]	0.55 [0.54 to 0.56]	0.66 [0.65 to 0.67]	0.68 [0.65 to 0.71]	0.30 [0.29 to 0.31]	0.44 [0.42 to 0.46]	0.78 [0.76 to 0.79]
F- <12 months	0.71 [0.69 to 0.72]	0.33 [0.32 to 0.33]	0.56 [0.54 to 0.57]	0.71 [0.70 to 0.72]	0.71 [0.68 to 0.74]]	0.32 [0.30 to 0.33]	0.50 [0.48 to 0.52]	0.78 [0.76 to 0.79]
F- <15 months	0.71 [0.69 to 0.72]	0.33 [0.32 to 0.33]	0.56 [0.54 to 0.57]	0.71 [0.70 to 0.72]	0.71 [0.68 to 0.74]	0.32 [0.30 to 0.33]	0.50 [0.48 to 0.52]	0.78 [0.76 to 0.79]
F- <18 months	0.71 [0.69 to 0.73]	0.33 [0.32 to 0.33]	0.53 [0.52 to 0.54]	0.71 [0.70 to 0.72]	0.71 [0.68 to 0.74]	0.30 [0.29 to 0.32]	0.55 [0.53 to 0.57]	0.74 [0.73 to 0.76]
F- <24 months	0.74 [0.73 to 0.76]	0.38 [0.38 to 0.39]	0.59 [0.58 to 0.61]	0.71 [0.70 to 0.73]	0.70 [0.67 to 0.73]	0.29 [0.28 to 0.30]	0.46 [0.44 to 0.48]	0.80 [0.78 to 0.81]
S- >20% RAND-BM diameter	0.62 [0.61 to 0.64]	0.15 [0.15 to 0.15]	0.47 [0.46 to 0.48]	0.72 [0.71 to 0.73]	0.57 [0.54 to 0.59]	0.13 [0.13 to 0.14]	0.60 [0.58 to 0.62]	0.76 [0.74 to 0.77]
S- > 10% volume	0.72 [0.70 to 0.73]	0.38 [0.37 to 0.39]	0.58 [0.57 to 0.59]	0.71 [0.70 to 0.72]	0.57 [0.54 to 0.59]	0.28 [0.27 to 0.29]	0.37 [0.35 to 0.38]	0.78 [0.76 to 0.80]
S- > 15% volume	0.74 [0.72 to 0.75]	0.41 [0.40 to 0.41]	0.60 [0.58 to 0.61]	0.73 [0.72 to 0.74	0.70 [0.67 to 0.73]	0.29 [0.28 to 0.30]	0.41 [0.39 to 0.43]	0.72 [0.70 to 0.74]
S- > 20% volume	0.74 [0.73 to 0.76]	0.38 [0.38 to 0.39]	0.59 [0.58 to 0.61]	0.71 [0.70 to 0.73]	0.70 [0.67 to 0.73]	0.29 [0.28 to 0.30]	0.46 [0.44 to 0.48]	0.80 [0.78 to 0.81]
S- > 25% volume	0.74 [0.73 to 0.76]	0.38 [0.38 to 0.39]	0.59 [0.58 to 0.61]	0.71 [0.70 to 0.73]	0.70 [0.67 to 0.73]	0.29 [0.28 to 0.30]	0.46 [0.44 to 0.48]	0.80 [0.78 to 0.81]
T-true progression	0.74 [0.73 to 0.76]	0.38 [0.38 to 0.39]	0.59 [0.58 to 0.61]	0.71 [0.70 to 0.73]	0.70 [0.67 to 0.73]	0.29 [0.28 to 0.30]	0.46 [0.44 to 0.48]	0.80 [0.78 to 0.81]
T-true and RN	0.67 [0.65 to 0.69]	0.33 [0.32 to 0.33]	0.60 [0.59 to 0.61]	0.64 [0.63 to 0.65]	0.67 [0.64 to 0.70]	0.40 [0.38 to 0.42]	0.53 [0.51 to 0.55]	0.69 [0.67 to 0.71]
T-true and PP	0.74 [0.72 to 0.76]	0.45 [0.44 to 0.45]	0.64 [0.63 to 0.65]	0.71 [0.70 to 0.72]	0.77 [0.74 to 0.80]	0.53 [0.51 to 0.55]	0.71 [0.69 to 0.72]	0.67 [0.65 to 0.69]
T-true and TRSC	0.70 [0.68 to 0.71]	0.42 [0.41 to 0.43]	0.63 [0.62 to 0.64]	0.66 [0.65 to 0.67]	0.72 [0.69 to 0.75]	0.55 [0.53 to 0.57]	0.64 [0.62 to 0.66]	0.64 [0.63 to 0.67]

**Fig. 10:**
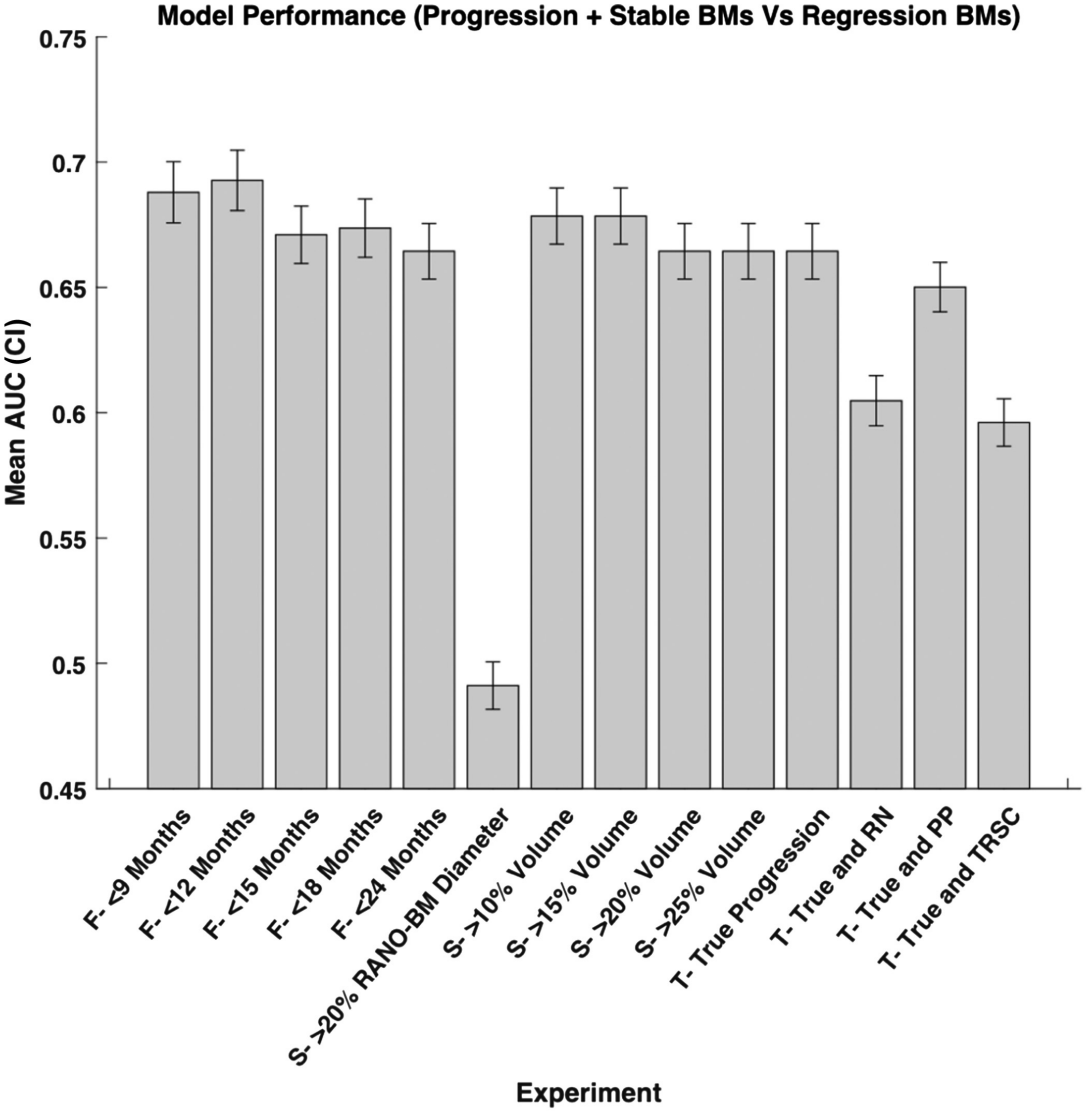
Experimental configurations we used to discern progressing and stable BMs versus regressing BMs and the corresponding model performance. The 95% confidence interval of each experiment is shown by the lines on the graph. CI = confidence interval, AUC = area under the receiver operating characteristic curve, True = true progression, RN = radiation necrosis, PP = pseudo-progression, and TRSC = treatment-related size changes. F = follow-up period category, S = size change metric category, and T = treatment-related size change category.

**Fig. 11:**
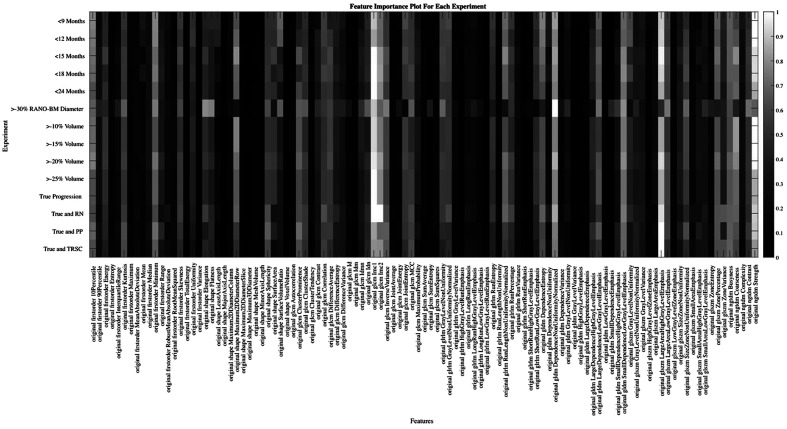
Depiction of the importance of each feature in each experiment predicting stable and progressing brain metastases versus regressing brain metastases. The row indicates which experiment is being referenced, and the column indicates what feature is being measured. Based on the color bar on the right of the image, the value of 1 (white color) indicates the feature was the most important for that experiment. The value of 0 (black color) indicates the feature was the least important for that experiment.

The performance of the models predicting progressing and stable BMs versus regressing BMs (as opposed to progressing BMs versus stable and regressing BMs) can be seen in Fig. 10 where the AUC range was 0.20 (0.49 to 0.69). The depiction of the feature importance for the progressing and stable BMs versus regressing BMs can be seen in Fig. 11.

Distinguishing stable and progressing BMs from regressing BMs produced lower model performance on average than classifying between progressing BMs and stable and regressing BMs. This lower performance is not a concern due lack of clinical relevance of the comparison of stable and progressing BMs compared with regressing BMs. Although the AUCs of 0.79^32^ and 0.87^30^ are strong predictors of the classification of stable and progressing BMs versus regressing BMs in the current literature, the lack of clinical relevance makes these models difficult to use in practice going forward.^33^

The similarity of the most important features for the progression and stable BM versus regressing BM comparison can be seen in Fig. 11. The similar pattern of feature importance differences for the experiments that classified between progressing and stable BMs and regressing BMs again emphasizes the hypothesis that the RANO-BM criteria cause larger changes to the labeling of the data set than other progression label types.

## Data Availability

The code used to generate the results can be freely accessed on GitHub (https://github.com/RobertPolicelli/PredictingBrainMetastasesProgression-SensitivityToChangingTheProgressionDefinition). The data that support the findings of this article are not publicly available due to the university research ethics board. They can be requested from the author at rpolicel@uwo.ca.

## References

[1] Achrol A. S. et al., “Brain metastases,” Nat. Rev. Dis. Primers 5(1), 5 (2019). 10.1038/s41572-018-0055-y 30655533

[2] Tsukada Y. et al., “Central nervous system metastasis from breast carcinoma. Autopsy study,” Cancer 52(12), 2349–2354 (1983). 10.1002/1097-0142(19831215)52:12<2349::AID-CNCR2820521231>3.0.CO;2-B 6640506

[3] Chakrabarty N. et al., “Imaging of brain metastasis in non-small-cell lung cancer: indications, protocols, diagnosis, post-therapy imaging, and implications regarding management,” Clin. Radiol. 78(3), 175–186 (2023). 10.1016/j.crad.2022.09.134 36503631

[4] Noh T. Walbert T., “Brain metastasis: clinical manifestations, symptom management, and palliative care,” Handb. Clin. Neurol. 149, 75–88 (2018). 10.1016/B978-0-12-811161-1.00006-2 29307363

[5] Gondi V. et al., “Radiation therapy for brain metastases: an ASTRO clinical practice guideline,” Pract. Radiat. Oncol. 12(4), 265–282 (2022). 10.1016/j.prro.2022.02.003 35534352

[r6] Vlachos N. et al., “Stereotactic radiosurgery versus whole-brain radiotherapy after resection of solitary brain metastasis: a systematic review and meta-analysis,” World Neurosurg. X 18, 100170 (2023). 10.1016/j.wnsx.2023.100170 36825221 PMC9942116

[7] Vogelbaum M. A. et al., “Treatment for brain metastases: ASCO-SNO-ASTRO guideline,” Neuro-Oncol. 24, 331–357 (2022). 10.1093/neuonc/noab262 34932393

[8] Churilla T. M. et al., “Comparison of local control of brain metastases with stereotactic radiosurgery vs surgical resection: a secondary analysis of a randomized clinical trial,” JAMA Oncol. 5(2), 243–247 (2019). 10.1001/jamaoncol.2018.4610 30419088 PMC6439566

[9] Asher A. L. et al., “Local failure after stereotactic radiosurgery (SRS) for intracranial metastasis: analysis from a cooperative, prospective national registry,” J. Neurooncol. 152(2), 299–311 (2021). 10.1007/s11060-021-03698-7 33481148

[10] DeVries D. A. et al., “Performance sensitivity analysis of brain metastasis stereotactic radiosurgery outcome prediction using MRI radiomics,” Sci. Rep. 12(1), 20975 (2022). 10.1038/s41598-022-25389-7 36471160 PMC9722896

[11] Jiang Z. et al., “Multimodality MRI-based radiomics approach to predict the posttreatment response of lung cancer brain metastases to gamma knife radiosurgery,” Eur. Radiol. 32(4), 2266–2276 (2022). 10.1007/s00330-021-08368-w 34978579

[r12] Mulford K. et al., “A radiomics-based model for predicting local control of resected brain metastases receiving adjuvant SRS,” Clin. Transl. Radiat. Oncol. 29, 27–32 (2021). 10.1016/j.ctro.2021.05.001 34095557 PMC8164004

[r13] Huang C. Y. et al., “Radiomics as prognostic factor in brain metastases treated with Gamma Knife radiosurgery,” J. Neurooncol. 146(3), 439–449 (2020). 10.1007/s11060-019-03343-4 32020474

[r14] Du P. et al., “Prediction of treatment response in patients with brain metastasis receiving stereotactic radiosurgery based on pre-treatment multimodal MRI radiomics and clinical risk factors: a machine learning model,” Front. Oncol. 13, 1114194 (2023). 10.3389/fonc.2023.1114194 36994193 PMC10040663

[r15] Lin N. U. et al., “Response assessment criteria for brain metastases: proposal from the RANO group,” Lancet Oncol. 16(6), e270–e278 (2015). 10.1016/S1470-2045(15)70057-4 26065612

[r16] Gutsche R. et al., “Radiomics outperforms semantic features for prediction of response to stereotactic radiosurgery in brain metastases,” Radiother. Oncol. 166, 37–43 (2022). 10.1016/j.radonc.2021.11.010 34801629

[r17] Ellingson B. M. et al., “Pseudoprogression, radionecrosis, inflammation or true tumor progression? Challenges associated with glioblastoma response assessment in an evolving therapeutic landscape,” J. Neurooncol. 134(3), 495–504 (2017). 10.1007/s11060-017-2375-2 28382534 PMC7893814

[r18] Parvez K. Parvez A. Zadeh G., “The diagnosis and treatment of pseudoprogression, radiation necrosis and brain tumor recurrence,” Int. J. Mol. Sci. 15(7), 11832–11846 (2014). 10.3390/ijms150711832 24995696 PMC4139817

[r19] Keek S. A. et al., “Predicting adverse radiation effects in brain tumors after stereotactic radiotherapy with deep learning and handcrafted radiomics,” Front. Oncol. 12, 920393 (2022). 10.3389/fonc.2022.920393 35912214 PMC9326101

[r20] Wang H. et al., “Predicting local failure of brain metastases after stereotactic radiosurgery with radiomics on planning MR images and dose maps,” Med. Phys. 48(9), 5522–5530 (2021). 10.1002/mp.15110 34287940

[r21] Karami E. et al., “Quantitative MRI biomarkers of stereotactic radiotherapy outcome in brain metastasis,” Sci. Rep. 9(1), 19830 (2019). 10.1038/s41598-019-56185-5 31882597 PMC6934477

[22] Liao C. Y. et al., “Enhancement of radiosurgical treatment outcome prediction using MRI radiomics in patients with non-small cell lung cancer brain metastases,” Cancers 13(16), 4030 (2021). 10.3390/cancers13164030 34439186 PMC8392266

[23] van Griethuysen J. J. M. et al., “Computational radiomics system to decode the radiographic phenotype,” Cancer Res. 77(21), e104–e107 (2017). 10.1158/0008-5472.CAN-17-0339 29092951 PMC5672828

[24] Saito T. Rehmsmeier M., “The precision-recall plot is more informative than the ROC plot when evaluating binary classifiers on imbalanced datasets,” PLoS One 10(3), e0118432 (2015). 10.1371/journal.pone.0118432 25738806 PMC4349800

[25] Efron B. Tibshirani R. J., An Introduction to the Bootstrap, 1st ed., Chapman and Hall/CRC (1994).

[26] Cha Y. J. et al., “Prediction of response to stereotactic radiosurgery for brain metastases using convolutional neural networks,” Anticancer Res. 38(9), 5437–5445 (2018). 10.21873/anticanres.12875 30194200

[27] Chukwueke U. N. Wen P. Y., “Use of the Response Assessment in Neuro-Oncology (RANO) criteria in clinical trials and clinical practice,” CNS Oncol. 8(1), CNS28 (2019). 10.2217/cns-2018-0007 30806082 PMC6499019

[28] Kawahara D. et al., “Predicting the local response of metastatic brain tumor to gamma knife radiosurgery by radiomics with a machine learning method,” Front. Oncol. 10, 569461 (2021). 10.3389/fonc.2020.569461 33505904 PMC7832385

[29] DeVries D. A. et al., “Dual-center validation of using magnetic resonance imaging radiomics to predict stereotactic radiosurgery outcomes,” [published correction appears in Neurooncol. Adv. 2023 Aug 11; 5(1), vdad093] Neurooncol. Adv. 5(1), vdad064 (2023). 10.1093/noajnl/vdad064 37358938 PMC10289521

[30] Jalalifar S. A. et al., “Impact of tumour segmentation accuracy on efficacy of quantitative MRI biomarkers of radiotherapy outcome in brain metastasis,” Cancers 14(20), 5133 (2022). 10.3390/cancers14205133 36291917 PMC9601104

[31] Ocaña-Tienda B. et al., “Volumetric analysis: rethinking brain metastases response assessment,” Neurooncol. Adv. 6(1), vdad161 (2023). 10.1093/noajnl/vdad161 38187872 PMC10771272

[32] Jaberipour M. et al., “A priori prediction of local failure in brain metastasis after hypo-fractionated stereotactic radiotherapy using quantitative MRI and machine learning,” Sci. Rep. 11, 21620 (2021). 10.1038/s41598-021-01024-9 34732781 PMC8566533

[33] Mouraviev A. et al., “Use of radiomics for the prediction of local control of brain metastases after stereotactic radiosurgery,” Neuro Oncol. 22(6), 797–805 (2020). 10.1093/neuonc/noaa007 31956919 PMC7283017

